# From gut dysbiosis to synaptic dysfunction in insomnia-associated depression: immune mechanisms and nutritional interventions

**DOI:** 10.3389/fimmu.2026.1940701

**Published:** 2026-10-20

**Authors:** Dan Tian, Hu Qi, Zhizhong Luo, Jili Xu, Jie Wu

**Affiliations:** 1 Chengdu University of Traditional Chinese Medicine, Chengdu, Sichuan, China; 2 Hospital of Chengdu University of Traditional Chinese Medicine, Chengdu, China

**Keywords:** depression, immune inflammation, insomnia, microbiota dysbiosis, microbiota-gut-brain axis, personalization, probiotics, synaptic plasticity

## Abstract

Insomnia and depression frequently coexist, yet their shared biological mechanisms have not been systematically integrated, limiting the development of targeted preventive and therapeutic strategies. Growing evidence identifies immune dysregulation as a key link between insomnia and depression, with the microbiota-gut-brain axis providing an important mechanistic framework. This review summarizes how insomnia induced gut microbiota alterations disrupt intestinal homeostasis, promote peripheral and central inflammation, and impair synaptic function during depression. It also discusses dietary interventions, food derived bioactive compounds, probiotics, and other microbiota targeted strategies. Targeting microbiota mediated immune pathways may provide new opportunities for the prevention and treatment of insomnia related depression.

## Graphical Abstract

**Figure:**
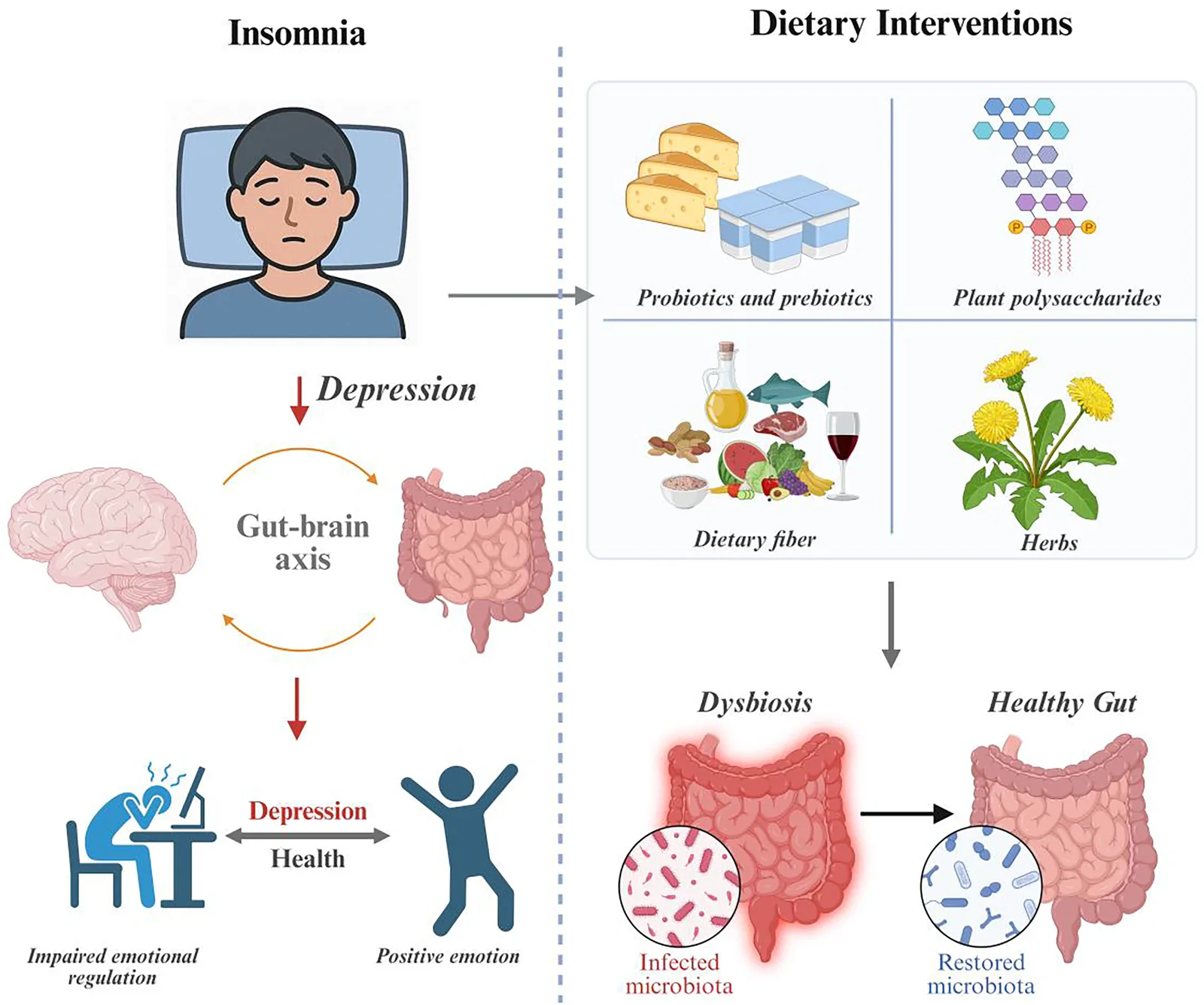
Gut microbiota dysbiosis associated with insomnia may contribute to immune dysregulation and depression. Interventions targeting the gut microbiota may help restore homeostasis between the gut and brain and alleviate depression associated with insomnia.

## 1 Introduction

Insomnia is one of the most common sleep-related disorders, with insomnia symptoms affecting approximately 35-50% of adults worldwide, although these estimates generally refer to symptoms rather than clinically diagnosed insomnia disorder (1–3). A prevalence of 64.6% has been reported in a specific population of Chinese adults based on questionnaire-defined sleep disturbances, rather than physician-diagnosed insomnia disorder, highlighting the substantial burden of sleep problems while requiring careful interpretation across different populations and diagnostic criteria (4). Depression is a highly disabling psychiatric disorder that affects hundreds of millions of people worldwide and imposes a substantial personal, social, and economic burden (5). Importantly, insomnia and depression frequently coexist, with insomnia symptoms observed in a large proportion of individuals with depression, and increasing evidence suggesting that insomnia represents not only a symptom but also an independent risk factor for depressive onset and recurrence (6–8).

Current mechanistic and therapeutic research on insomnia and depression has largely focused on circadian rhythm disruption, including interventions such as light therapy, melatonin administration, and chronotherapy (9). However, accumulating evidence indicates that the biological interaction between these disorders involves multiple interconnected systems beyond circadian regulation. Among these, the microbiota–gut–brain axis has emerged as an important framework linking sleep disturbance, immune dysfunction, and neuropsychiatric disorders. The gut microbiota communicates bidirectionally with the central nervous system through microbial metabolites, immune signaling, neuroendocrine pathways, and neural routes, forming the microbiota-gut-brain axis (10). Dietary factors and food-derived bioactive compounds can further influence this network by modifying microbial composition, metabolite profiles, intestinal barrier function, and host immune responses.

Altered gut microbial composition has been reported in both insomnia and depression, although microbial signatures specific to their comorbidity remain incompletely characterized. Emerging evidence suggests that insomnia-associated gut dysbiosis may impair intestinal barrier integrity, promote peripheral and central inflammatory activation, and contribute to neuronal dysfunction and impaired synaptic plasticity (11). Nevertheless, the mechanisms linking microbiota-mediated immune alterations with depressive phenotypes remain insufficiently integrated, particularly from the perspective of nutritional regulation and microbiota-targeted intervention.

Previous reviews have addressed the microbiome in relation to insomnia, depression, and related neurobiological mechanisms (12, 13). Building on this work, the present review focuses specifically on depression associated with insomnia and integrates the pathological continuum from gut microbial dysregulation and intestinal barrier dysfunction to immune activation, neuroinflammation, and impaired synaptic plasticity within a microbiota immune synapse framework. We further examine dietary interventions, food derived bioactive compounds, probiotics, and other microbiota and immune targeted strategies, while distinguishing their supporting levels of evidence and translational relevance. This integrated perspective aims to provide a comprehensive basis for understanding the pathophysiology of insomnia associated depression and for identifying potential nutritional and therapeutic approaches.

## 2 Gut microbiota brain axis in the context of insomnia-associated depression

The balance of gut microbiota is closely linked to human health, and its association with depression has emerged as a promising target in mental health interventions (14). Insomnia, a common sleep disorder, may disrupt gut microbial homeostasis through the bidirectional microbiota-gut-brain axis, thereby influencing overall health (15). Sleep disturbances and gut dysbiosis exhibit a bidirectional relationship: sleep deprivation perturbs gut microbiota composition and metabolism (16), The relationship between sleep disturbances and gut dysbiosis is bidirectional: sleep deprivation alters the composition and function of gut microbiota, while microbial imbalance may in turn impair sleep quality. These findings suggest that gut dysbiosis may be a key contributor to sleep disorders (17). Advances in microbiota-gut-brain axis research have highlighted microbiota-targeted functional compensatory therapeutics, including probiotics, prebiotics, small molecules, plant polysaccharides, and synthetic biology-engineered microbes (18). The relationship between the microbiota and insomnia-associated depression is shown in **Figure 1**. By enhancing beneficial taxa and preserving microbial homeostasis, these strategies promote host health with reduced adverse effects and improved adherence.

**Figure 1:**
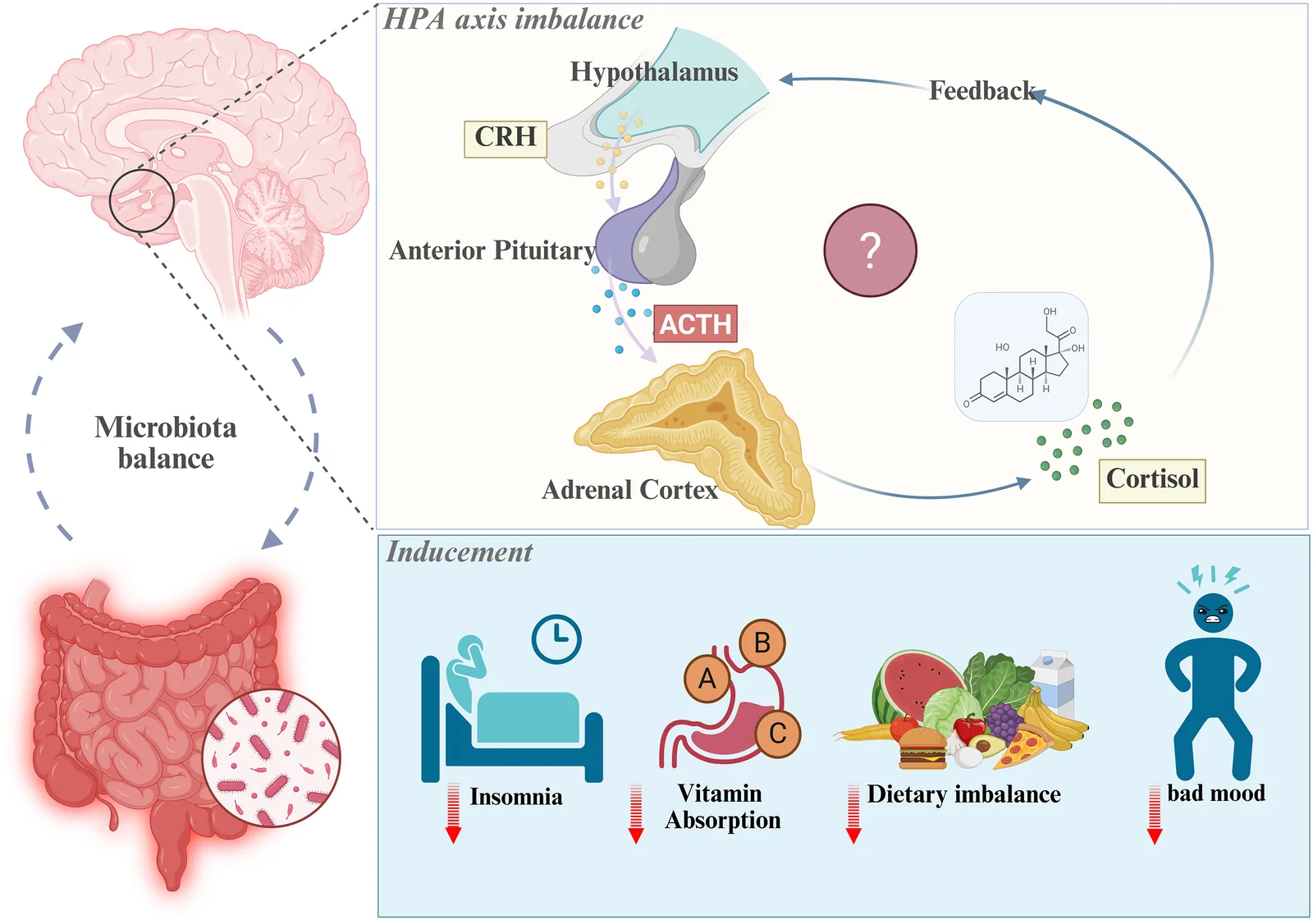
Nutritional and physiological triggers of the microbiota brain axis and HPA axis imbalance. Insomnia and dietary imbalance compromise vitamin absorption and disrupt gut microbial homeostasis. These factors activate the hypothalamus pituitary adrenal (HPA) axis, initiating a cascade where hypothalamic CRH and pituitary ACTH stimulate adrenal cortisol secretion. The resulting endocrine feedback loop links intestinal dysbiosis to central nervous system dysfunction, providing a mechanistic pathway for the progression from sleep disturbance to mood disorders.

### 2.1 Constituents of the gut microbiota brain axis and their relevance to insomnia-associated depression

The microbiota-gut-brain axis exerts its effects primarily through the vagus nerve, endocrine system, immune system, and metabolic pathways (11). The vagus nerve, the longest cranial nerve connecting the peripheral and central nervous systems, serves as a critical conduit for signal transmission and physiological regulation within this axis (19). Emerging evidence indicates that gut microbiota can produce neurotransmitters such as acetylcholine and dopamine, which influence emotional, behavioral, and cognitive functions via vagal signaling (20). The microbiota-gut-brain axis also involves endocrine pathways, notably the HPA axis (21).

Gut microbiota can modulate host stress responses by influencing HPA axis activity. As a central hub of gut-brain communication, the HPA axis is initiated by the release of corticotropin-releasing hormone (CRH) from the paraventricular nucleus of the hypothalamus (22), which stimulates the pituitary to secrete adrenocorticotropic hormone (ACTH), subsequently promoting cortisol production by the adrenal cortex. Cortisol then exerts negative feedback on both CRH and ACTH to maintain homeostasis. Notably, the secretion of these hormones is regulated by circadian rhythms and occurs in parallel with gut microbiota development (23). Immune signaling, particularly cytokine-mediated pathways, represents another key mechanism through which the microbiota-gut-brain axis influences the central nervous system. Gut microbiota modulates neuroimmune communication by producing short-chain fatty acids (SCFAs), lipopolysaccharides (LPS), and pro-inflammatory cytokines such as IL-1β, IL-6, and TNF-α. These mediators can affect Blood-Brain Barrier (BBB) permeability, alter vagal nerve signaling, and modulate HPA axis activity, thereby impacting central nervous system function (24). The following is the corresponding logical mechanism pathway **Figure 2**.

**Figure 2:**
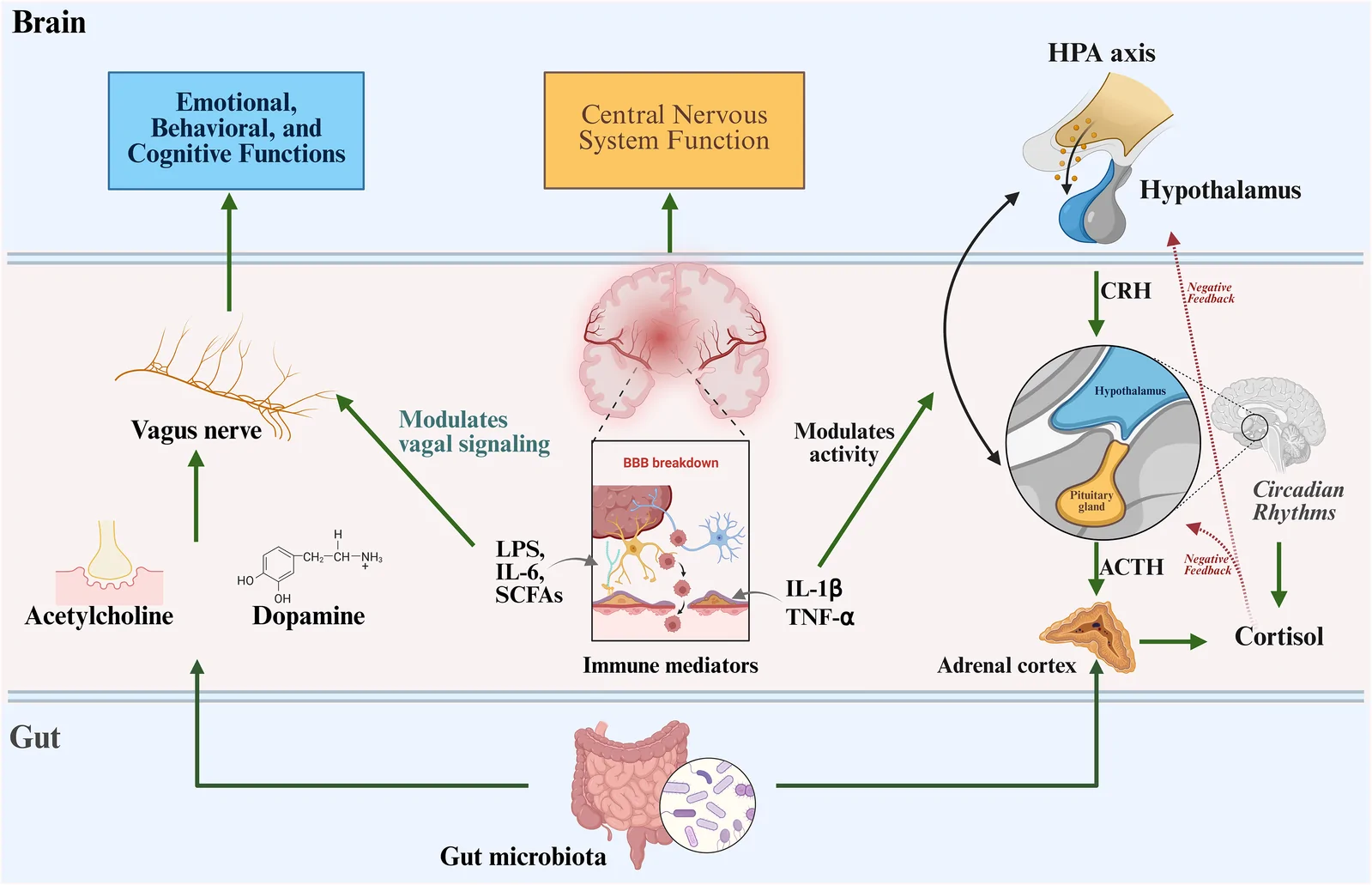
Gut microbiota–brain axis mechanisms linking gut derived signals to insomnia-associated depression. Gut microbiota communicates with the brain through vagal, immune and endocrine pathways. Microbiota-derived neurotransmitters, including acetylcholine and dopamine, modulate emotional, behavioral and cognitive functions via the vagus nerve, whereas LPS, SCFAs and pro-inflammatory cytokines influence blood–brain barrier integrity, central nervous system function and HPA axis activity. Circadian rhythmicity and cortisol feedback further integrate these signals, collectively contributing to the pathophysiology of insomnia-associated depression.

### 2.2 Alterations in gut microbiota composition and function in insomnia-associated depression

Insomnia markedly reduces the α-diversity of the gut microbiota, characterized by an imbalanced Firmicutes-to-Bacteroidetes ratio (25). This is accompanied by a decreased abundance of beneficial genera such as *Faecalibacterium* and *Roseburia* (26), alongside an increased relative abundance of opportunistic taxa including *Prevotella* and *Blautia* (27). Such compositional shifts primarily disrupt the tryptophan metabolic pathway, leading to reduced 5-hydroxytryptamine (5-HT, serotonin) synthesis and consequently impairing the microbiota-gut-brain axis in emotional regulation (28).

Insomnia is often accompanied by elevated cortisol levels, which, via glucocorticoid receptor (GR) activation, suppress the expression of key tight junction proteins ZO-1, occludin, and Claudin-1 in intestinal epithelial cells (29), thereby compromising gut barrier integrity and inducing a “leaky gut” phenotype (30). The resulting barrier disruption increases translocation of pathogen-associated molecular patterns (PAMPs), such as LPS, into the circulation, activating the TLR4/NF-κB inflammatory pathway (31). This, in turn, facilitates peripheral cytokines including IL-6, TNF-α, and IL-1β to penetrate the BBB, exacerbating microglial activation, impairing neuroplasticity, and ultimately contributing to depression onset. Therapeutic strategies aimed at reconstructing microbial metabolic pathways, restoring gut barrier function, and alleviating systemic inflammation hold promise for overcoming the single target limitations of conventional antidepressants (32). Targeting the “microbiota-immune-synapse” axis to achieve system level modulation represents a key direction in the development of next generation antidepressant interventions. The following is the corresponding logical mechanism pathway **Figure 3**. We have compiled clinical cases featuring the characteristics of gut microbiota imbalance in depression following insomnia, as shown in **Table 1**.

**Figure 3:**
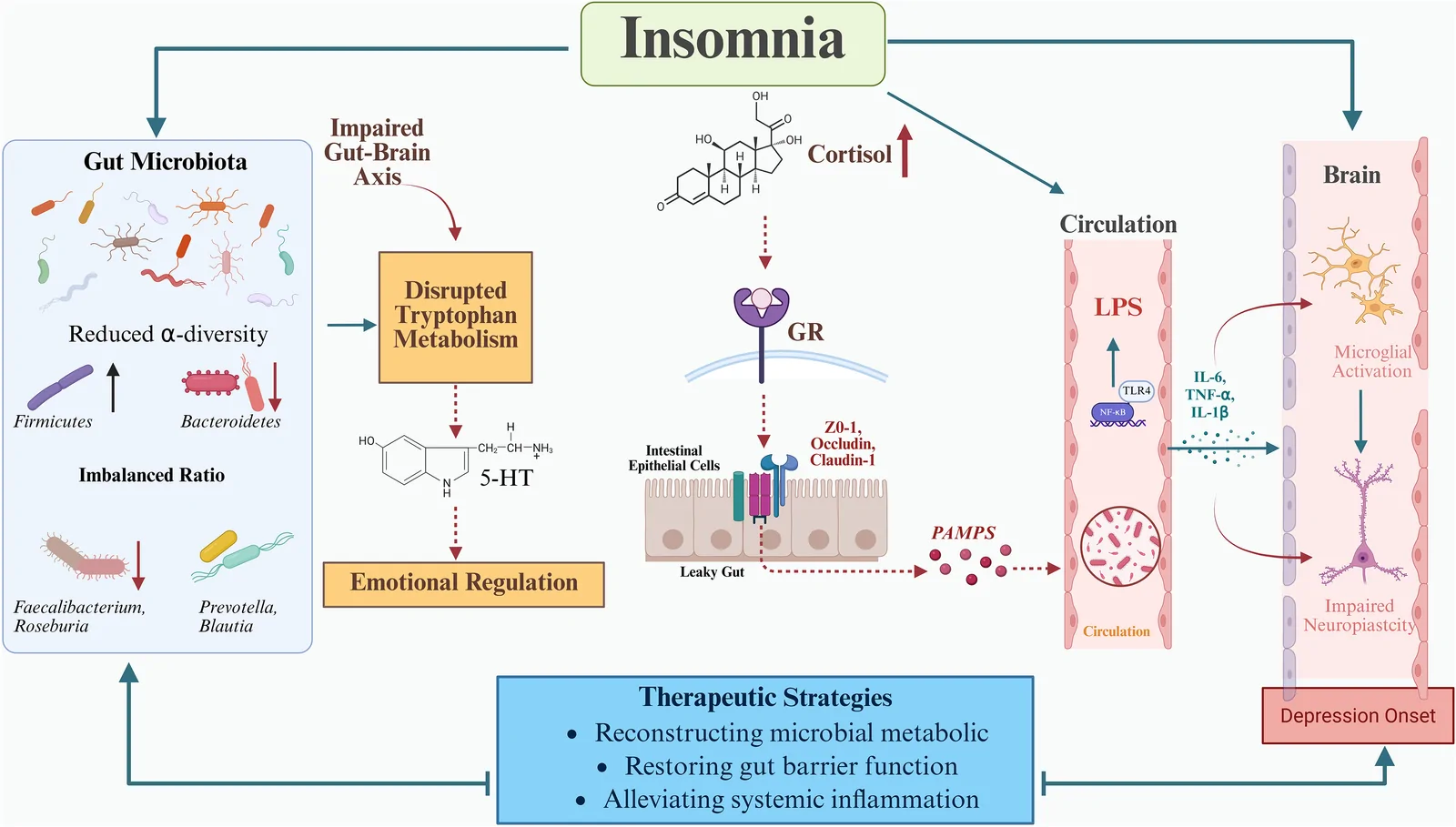
Gut microbiota dysbiosis links insomnia to depression. Insomnia reduces gut microbial α-diversity, disrupts the Firmicutes-to-Bacteroidetes balance and alters key taxa, thereby impairing tryptophan metabolism and 5-HT-dependent emotional regulation. Concurrent cortisol-GR signaling damages intestinal barrier integrity, promotes LPS/PAMP translocation and peripheral inflammation, and facilitates BBB dysfunction, microglial activation and impaired neuroplasticity, ultimately contributing to depression onset.

**Table 1:** Microbial imbalance characteristics associated with insomnia and depression.

Subjects	Type	Function	Depression	Abnormal flora	References
Human (n=24)	Chronic insomnia	SCFA production, immune regulation, anti-inflammation	No	*↓ Bacteroidaceae, ↓ Ruminococcaceae, ↓ Bacteroides, ↑ Prevotellaceae, ↑ Prevotella, ↑ Gemmiger, ↑ Fusicatenibacter*	(33)
Human (n=20 Acute, n=38 Chronic)	Acute and Chronic insomnia	SCFA production, anti-inflammation	No	Acute:*↓ Lachnospira, ↓ Bacteroides*, Chronic:*↓ Faecalibacterium, ↓ Roseburia, ↑ Blautia, ↑ Eubacterium hallii*	(34)
Female mice	Chronic Sleep deprivation	SCFA production, anti-inflammatory, intestinal regulation	Yes	*↑ Rothia, ↑ Streptococcus, ↑ Pantoea, ↑ Klebsiella, ↓ Rikenella, ↓ Eubacterium xylanophilum group, ↓ Eisenbergiella*	(35)
Human (n=36)	Chronic insomnia	SCFA production, anti-inflammation	Yes	*↑ Dorea, ↑ Intestinibacter, ↓ Coprococcus*	(36)
Human (n=40)	Chronic insomnia	Promotes sleep, anti-inflammatory	Yes	*↑ Bacteroides, ↓ Bifidobacterium*	(37)
Human (n=20)	Chronic insomnia	SCFA production, anti-inflammatory, sleep promotion	No	*↑ Prevotella, ↑ Gemmiger, ↑ Fusicatenibacter, ↓ Bacteroides, ↓ Coprococcus, ↓ Oscillibacter, ↓ Clostridium XI*	(33)
Human (n=38)	Chronic insomnia	SCFA production, anti-inflammation	No	*↓ Faecalibacterium, ↓ Roseburia, ↑ Blautia*	(34)
Human (n=45)	Chronic insomnia	SCFA production, anti-inflammation	Yes	*↑ Dorea, ↑ Intestinibacter, ↓ Coprococcus*	(36)
Human (n=159)	Sleep quality varying	Microbial Community Structure and Sleep Quality Link	Yes	*↑ F. prausnitzii*	(38)

↑, increased abundance; ↓, decreased abundance.

### 2.3 Gut microbiota-mediated modulation of tryptophan metabolism in sleep disturbance-related depression

Tryptophan, an essential amino acid, plays a pivotal role in regulating sleep-wake rhythms. It is metabolized through multiple pathways to produce neuroactive compounds (39), primarily including 5-HT, kynurenine, and various indole derivatives (40). Among these, the 5-HT-melatonin pathway is most directly associated with sleep quality.

5-HT biosynthesis primarily depends on the activity of tryptophan hydroxylase isoforms TPH1 and TPH2, which catalyze the conversion of tryptophan into 5-hydroxytryptophan (5-HTP) (41). 5-HTP is then converted to 5-HT by aromatic L-amino acid decarboxylase (AADC), and subsequently transformed in the pineal gland into melatonin-an essential hormone for circadian rhythm regulation and sleep initiation (42). Studies indicate that in insomnia-associated depression, exacerbated neuroinflammation serves as an upstream driver that downregulates central TPH2 expression. This suppression consequently may leads to reduced 5-HT and melatonin levels, which further aggravates insomnia symptoms. Such biochemical shifts are closely linked to alterations in gut microbiota composition, particularly within the phylum Firmicutes (43).

Dysbiosis of the gut microbiota can alter tryptophan metabolism, thereby affecting the production and function of 5-HT. For instance, specific genera such as *Lactobacillus* and *Bifidobacterium* can upregulate TPH1 expression, enhancing 5-HT synthesis and exerting antidepressant effects (44, 45). Notably, gut microbiota composition, metabolic activity, and stress resilience vary considerably across individuals depending on age, sex, and environmental factors, which may explain the suboptimal efficacy of single-target interventions (46). Developing “microbiota function compensating” therapeutics based on the metabolism-transport-neuro regulation network, along with microbiome modulation through probiotics or dietary interventions, represents a promising strategy to alleviate insomnia, restore neurotransmitter homeostasis, and optimize sleep architecture. The following is the corresponding logical mechanism pathway **Figure 4**.

**Figure 4:**
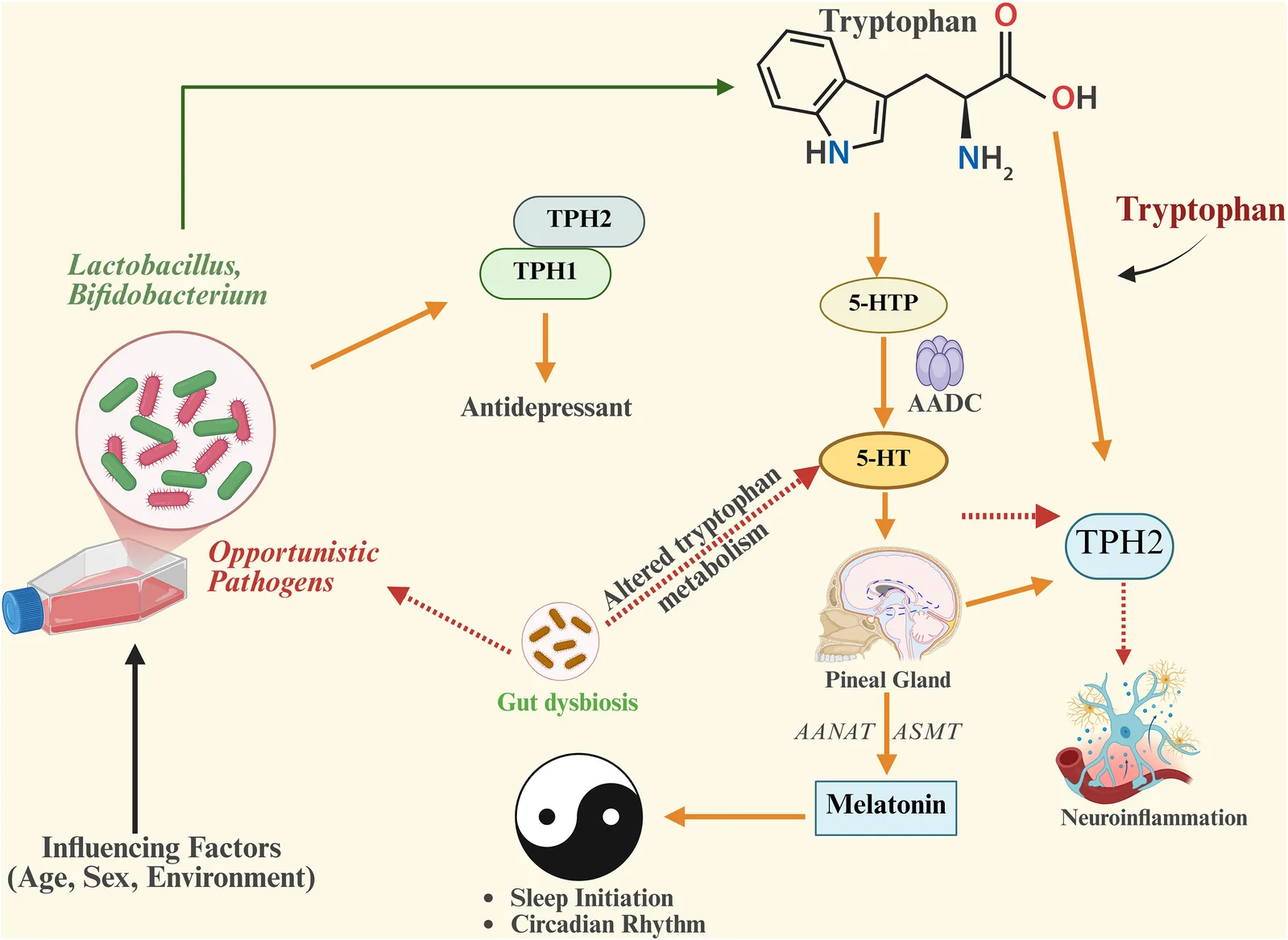
Microbial regulation of the tryptophan serotonin metabolic pathway and neuroinflammation. Host factors shape gut microbiota composition, with beneficial taxa such as Lactobacillus contributing to tryptophan metabolism. Tryptophan is converted by TPH1 and TPH2 into 5-HTP and 5-HT to maintain pineal gland function and circadian rhythms. Conversely, opportunistic pathogens and dysbiosis disrupt 5-HT metabolism and trigger TPH2-mediated neuroinflammation. This framework suggests how microbial homeostasis promotes antidepressant effects while dysbiosis drives neuroinflammatory sleep and mood disturbances.

### 2.4 Role of gut-derived GABA in neurophysiology of insomnia-associated depression

γ-Aminobutyric acid is a key inhibitory neurotransmitter that regulates neuronal excitability, facilitating relaxation and the initiation of sleep (47). Recent studies indicate that the gut microbiota plays a crucial role in GABA metabolism, and targeting this metabolic cycle offers valuable insights into the pathophysiology and treatment of insomnia-associated depression (48). Genera such as Bacillus and *Lactobacillus* can produce GABA or activate GABA receptors to enhance sleep propensity. Patients with insomnia and depression often exhibit reduced GABA levels in the brain, along with structural impairments in α1, β2, and γ2 subunits of GABA receptors, leading to diminished inhibitory synaptic signaling and neuronal excitability (49). Restoring microbial balance to boost GABA biosynthetic enzymes, particularly glutamate decarboxylase (GAD) activity, may help ameliorate such neural damage. The regulatory relationship of the brain-gut axis is shown in **Figure 5**. Additionally, oral GABA supplementation and the development of delivery systems with improved bioavailability represent promising microbiota-gut-brain axis-based strategies for indirectly modulating central GABA pathways to alleviate insomnia-associated depression.

**Figure 5:**
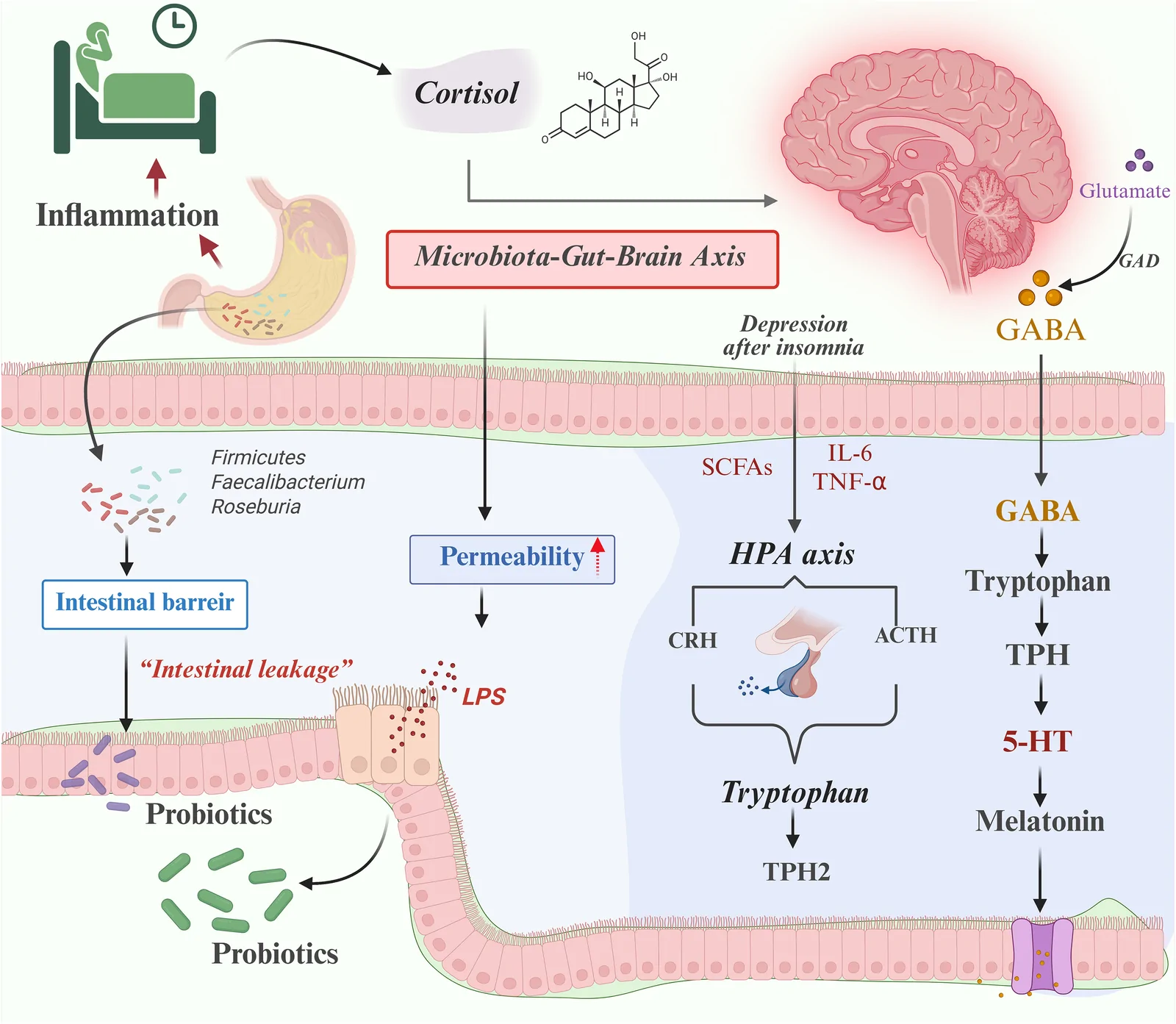
Insomnia affects depression through the microbiota axis. Insomnia disrupts circadian rhythm and activates the HPA axis, leading to cortisol elevation and intestinal inflammation. These changes impair gut barrier integrity and increase permeability, which facilitates LPS translocation and microbial dysbiosis characterized by reduced production of short-chain fatty acids and GABA. Tryptophan metabolism is reprogrammed, shifting serotonin and melatonin synthesis, and causing imbalance between excitatory glutamate and inhibitory GABA signaling.

## 3 Immune axis imbalance in the pathogenesis of insomnia-associated depression

With growing research into the interplay between sleep and immunity, accumulating evidence reveals a complex bidirectional relationship between insomnia-associated depression and the immune system (50). This dynamic regulatory network is closely linked to multiple neurotransmitters, cytokines, and HPA axis activity (51). Immune imbalance may manifest as either immunosuppression or excessive inflammatory responses, depending on the duration and severity of insomnia, as well as individual variability.

Insomnia reduces the activity of natural killer cells, T cells, and phagocytes, thereby weakening host defense capacity (52). With compromised immune integrity, these cells become less efficient in detecting and eliminating pathogens, cancer cells, and damaged cells, increasing susceptibility to infections and disease. The immune system also interacts with the central nervous system (CNS) via pro-inflammatory signaling to influence melatonin secretion, thereby regulating the sleep and wake cycle (53). Under physiological conditions, IL-1 and TNF-α key mediators of innate immunity act as “sleep molecules” by modulating non-rapid eye movement (NREM) sleep and maintaining sleep quality (54). The following is the corresponding logical mechanism pathway **Figure 6**. In contrast, insomnia is often accompanied by excessive secretion of pro-inflammatory cytokines, driving chronic neuroinflammation and contributing to the pathogenesis of multiple disorders, including cardiovascular disease, diabetes, and neurodegenerative conditions (55).

**Figure 6:**
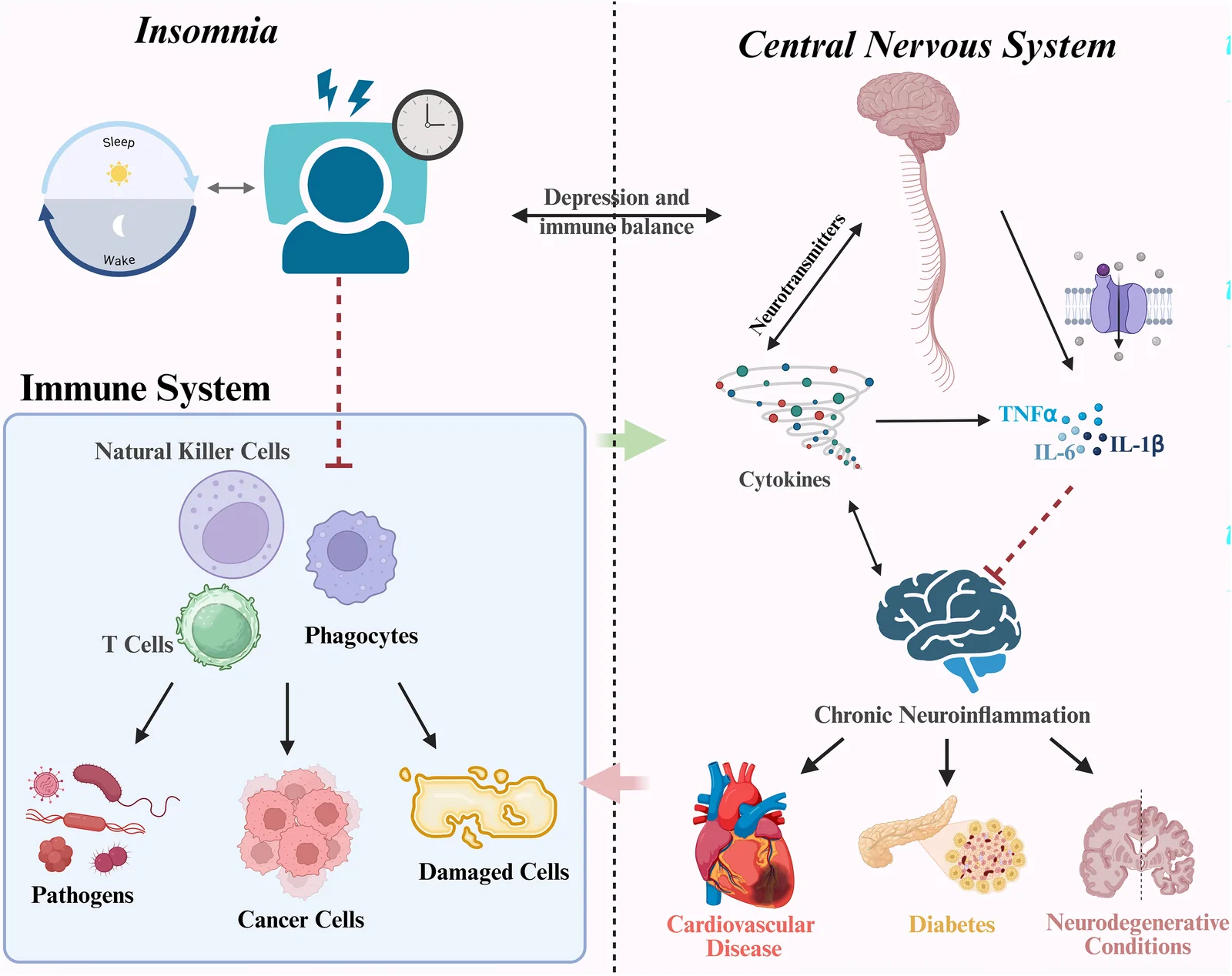
Immune-brain crosstalk in insomnia-associated depression. Insomnia disrupts sleep homeostasis and impairs immune surveillance by reducing the activity of natural killer cells, T cells and phagocytes. At the same time, increased pro-inflammatory mediators, including TNF, IL-6 and IL-1β, promote neuroinflammation through immune–brain communication pathways, disturb melatonin-related sleep regulation and contribute to depressive symptoms. This sustained neuroimmune imbalance may also increase the risk of systemic complications, including cardiovascular, metabolic and neurodegenerative disorders.

### 3.1 The association between insomnia-induced inflammatory cytokine storms and depression

As described above in Section 2.2, stress-induced HPA-axis activation and elevated cortisol may impair intestinal barrier integrity, facilitating microbial product translocation and subsequent immune activation. This process involves TLR4/NF-κB signaling, inflammatory cytokine release, and downstream effects on BBB integrity and microglial activation. The imbalance of inflammatory factors also promotes activation of the NOD-like receptor protein 3 (NLRP3) inflammasome, inducing neuronal apoptosis and impairing synaptic function (56). Moreover, pro-inflammatory cytokines TNF-α and IL-1β suppress the expression of brain-derived neurotrophic factor (BDNF), postsynaptic density protein 95 (PSD-95) (57), and synaptophysin by activating the MAPK signaling pathway, resulting in reduced synaptic plasticity and consequently leading to cognitive and emotional disorders (58). Developing targeted therapies that stabilize immune function and regulate inflammation following insomnia is a critical strategy for treating depression caused by chronic sleep disturbances.

### 3.2 The role of prostaglandin D_2_ as a bridge between sleep and immunity

Prostaglandin D**_2_** (PGD**_2_**), a metabolite of arachidonic acid, has been proposed as a mediator linking sleep regulation and peripheral immune responses (59). Under sleep deprivation, increased activity of lipocalin-type prostaglandin D synthase (L-PGDS) in the meninges and glial cells leads to a significant elevation of PGD**_2_** levels (60). Transported across the BBB into the peripheral circulation via MRP4, PGD**_2_** disrupts peripheral immune homeostasis. However, the proposed PGD2-mediated inflammatory pathway is supported primarily by mechanistic and animal studies, and its relevance to human insomnia-associated depression remains to be established.

In the periphery, PGD**_2_** binds to the high-affinity receptor GPR44, activating the Gs protein and stimulating adenylyl cyclase to produce cAMP (61). This cascade further activates protein kinase A (PKA) and the CREB pathway, regulating the expression of immune cell functional genes. Neutrophils, eosinophils, and Th2 cells are notably activated, promoting their migration to peripheral tissues and releasing pro-inflammatory cytokines such as IL-4, IL-5, IL-13, and TNF-α, thereby exacerbating immune imbalance (62). Studies have shown that specific antagonism of the DP1 receptor or inhibition of PGD**_2_** synthase expression effectively suppresses neutrophil migration and reduces peripheral inflammatory cytokines, reversing sleep deprivation-induced systemic immune activation and alleviating depression-related symptoms (63). The key immune factor regulatory mechanism of insomnia-associated depression is shown in **Figure 7**.

**Figure 7:**
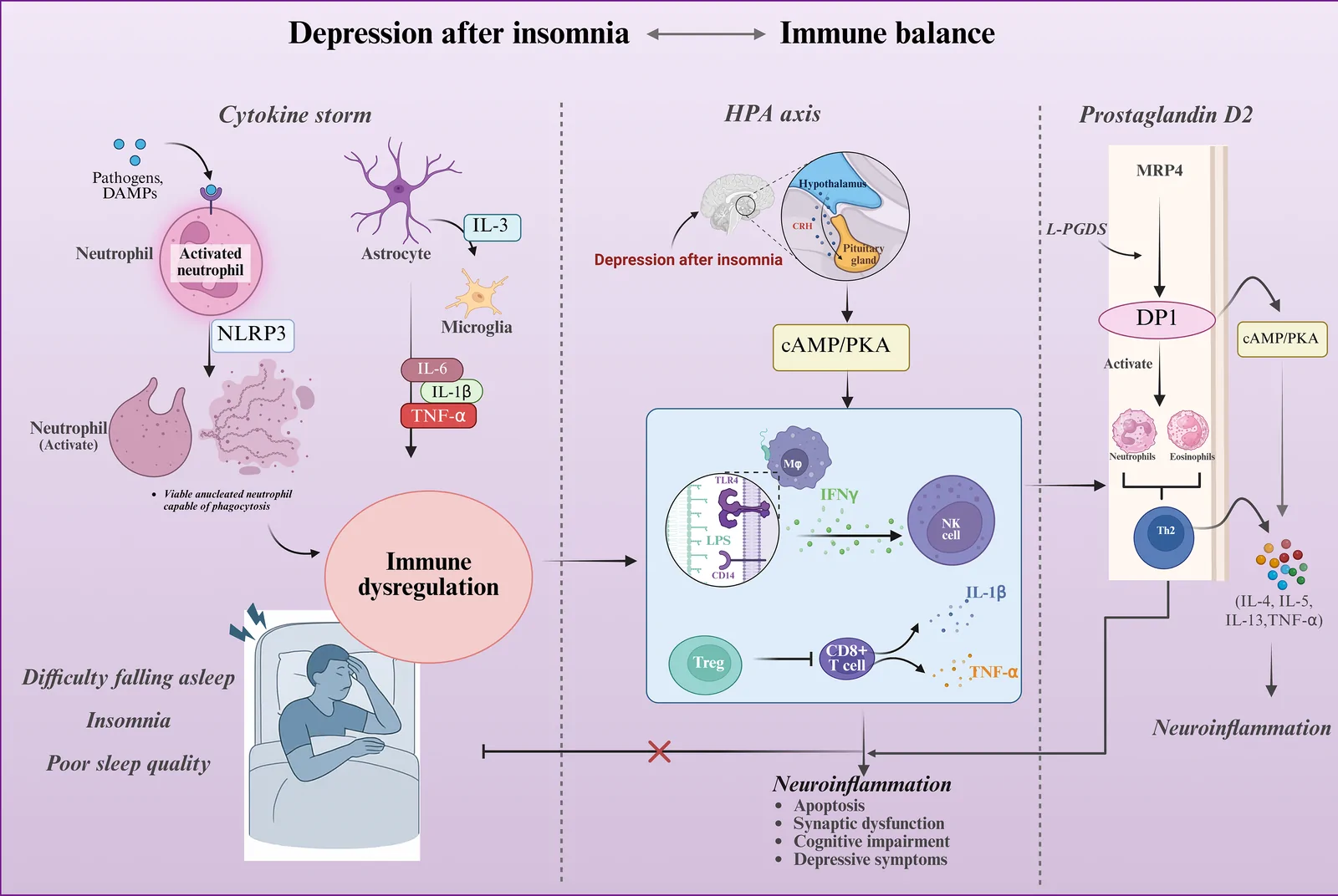
Immune axis in insomnia-related depression. Insomnia activates peripheral neutrophils and assembles the NLRP3 inflammasome, resulting in increased secretion of proinflammatory cytokines such as IL-1β, IL-6, TNF-α, and IL-3, which in turn activate microglia and astrocytes. Simultaneously, the HPA axis modifies immune homeostasis through the cAMP–PKA pathway, with reduced regulatory T cells and enhanced effector T-cell activity, thereby amplifying neuroinflammation and synaptic dysfunction. PGD**_2_** signaling via DP1 receptors and cAMP–PKA, as well as MRP4-related pathways, further augment inflammatory responses and contribute to depressive outcomes.

### 3.3 Persistent HPA axis activation in insomnia-induced depression

The sustained activation of the HPA axis is considered a central neuro-immune-endocrine pathway in the pathogenesis of insomnia-induced depression (64). Chronic stress from insomnia stimulates the excitability of CRH neurons in the paraventricular nucleus (PVN) of the hypothalamus (65), leading to increased synthesis and secretion of CRH. CRH acts on CRHR1 receptors in the anterior pituitary, activating the adenylyl cyclase (AC)/cAMP/PKA signaling pathway, resulting in elevated cortisol levels (66). Under sustained HPA axis hyperactivation, high cortisol concentrations suppress transcription of pro-inflammatory cytokines such as IL-1β, IL-6, and TNF-α, inhibit T cell proliferation, Th1 cell activation, and IFN-γ expression, while promoting a shift toward Treg and Th2 cells (67). This shift weakens cellular immune responses and impairs antigen clearance capacity.

Chronic insomnia may also lead to glucocorticoid receptor overload and the development of glucocorticoid resistance (68), weakening the negative feedback regulation on inflammatory responses (69). This results in sustained elevation of IL-6 and TNF-α, establishing a chronic low-grade inflammatory state that suppresses the expression of BDNF within the central nervous system (70). Ultimately, this cascade contributes to the development of a depressive phenotype characterized by mood disturbances and cognitive impairment. Representative pharmacological agents targeting immune pathways and the HPA axis are summarized in **Table 2**, together with their levels of evidence and potential relevance to depression associated with insomnia.

**Table 2:** Targeted drugs that improve insomnia-associated depression by regulating immunity.

Drug name	Effect	Target	Key pathways	Dosage	Evidence level	Reference
Asapiprant	Asthma, allergic rhinitis, inflammation	PGD2 receptors	PGD2-DP1/DP2 axis, cytokine response	100 mg/day	PE	(71)
Prednisone	Inflammation, autoimmune diseases	Glucocorticoid receptor	HPA axis, T cell and cytokine suppression	5–60 mg/day	OHE	(72)
Fluoxetine	Depression	5-HT reuptake inhibition, HPA axis	HPA axis modulation via serotonergic	20–80 mg/day	PE	(73)
Ketoconazole	Fungal infections, endocrine disorders	Cytochrome P450 enzymes, steroidogenesis	Steroid biosynthesis inhibition, HPA axis	200–400 mg/day	OHE	(74)
DHEA	Endocrine dysfunction	Glucocorticoid, androgen receptors	Steroid hormone modulation, neuroimmune balance	25–100 mg/day	OHE	(75)
Ibuprofen	Pain and inflammation	Cyclooxygenase (COX)	COX-PGE2, neuroinflammation	200–400 mg every 4–6 h	PE	(76)
Loratadine	Allergic diseases	Histamine H1 receptor antagonist	Allergic inflammation, histamine	10 mg/day	OHE	(77)
Cyclosporine	Autoimmune diseases	Calcineurin inhibition	T cell signaling, NFAT	2.5–5 mg/kg/day	PE	(78)
Infliximab	Ankylosing spondylitis, depression, anxiety	TNF-α	TNF-α-mediated inflammatory signaling	5 mg/kg every 8 weeks	OHE	(79)
Tocilizumab	Inflammatory diseases	IL-6 receptor blockade	IL-6-JAK-STAT3	8 mg/kg every 4 weeks	OHE	(80)
Thalidomide	Multiple myeloma, lymphoma	Cereblon modulation	TNF-α and CRBN-mediated immune modulation	10 mg/day	PE	(81)

PE, preclinical evidence; OHE, observational human evidence; RCE, randomized clinical evidence; H/PM, hypothesis or proposed mechanism.

## 4 Synaptic axis: central role in neural communication and emotional stability

The synaptic axis, as a central regulator of neuronal communication, emotional stability, and cognitive function, relies heavily on the integrity of synaptic structure and the dynamic regulation of synaptic plasticity (82).

Studies have shown that chronic sleep deprivation significantly impairs synaptic plasticity, primarily long-term potentiation (LTP), especially in emotion-regulating brain regions such as the hippocampal CA1 area, prefrontal cortex, and amygdala (83). This impairment is mainly associated with a marked downregulation of BDNF expression and limited activation of its receptor TrkB signaling axis (84). Additionally, excessive release of the excitatory neurotransmitter glutamate induces intracellular Ca^2+^ overload, causing excitotoxicity that ultimately leads to synaptic damage and neuronal apoptosis (85). Moreover, insomnia weakens GABAergic function by downregulating GABA receptor subunits (α1, β2, γ2), resulting in overall neural network hyperexcitability and disrupted neuronal firing patterns, which contribute to anxiety and depressive behaviors (86).

### 4.1 The mechanism of insomnia’s damage to synaptic plasticity

Synaptic plasticity is a fundamental physiological process essential for maintaining emotional stability and cognitive function, and it is closely linked to sleep quality and states. Synaptic plasticity encompasses three main aspects: modulation of synaptic strength, reversible regulation of synaptic structure, and synaptic function (87). Key processes involved include LTP, long-term depression (LTD), dendritic spine remodeling, and post-synaptic receptor activity (88). Slow-wave sleep (SWS) and rapid eye movement (REM) sleep represent critical temporal windows for synaptic plasticity, primarily consolidating synaptic stability to enhance neural network information processing efficiency and emotional regulation (89).

Studies have shown that in chronic sleep restriction (CSR) animal models, there is a significant downregulation of PSD-95 expression, thinning of the postsynaptic density, and a reduction in dendritic spine density. Additionally, key synaptic receptors such as GluA1 and NR2B are suppressed (90). These synaptic alterations mirror the deficits observed in depression models induced by chronic unpredictable mild stress (CUMS), suggesting a close link between insomnia-induced synaptic damage and the pathogenesis of depression (91). Prolonged sleep deprivation enhances glutamate excitotoxicity, causing excessive Ca^2+^ influx through NMDA receptors, which activates caspase cascades leading to reactive oxygen species (ROS) production and oxidative damage to synaptic structures (92). Concurrently, impaired GABA function reduces inhibitory synapses, disrupting the excitation-inhibition balance within neural networks and exacerbating depressive symptoms (93). Therefore, given that sleep is essential for maintaining synaptic plasticity, future therapeutic strategies targeting insomnia-associated depression should focus on restoring synaptic homeostasis and functional consolidation by regulating synaptic protein synthesis, receptor trafficking, dendritic spine remodeling, and neurotrophic factor signaling. The relationship between insomnia-associated depression and synaptic axes is shown in **Figure 8**.

**Figure 8:**
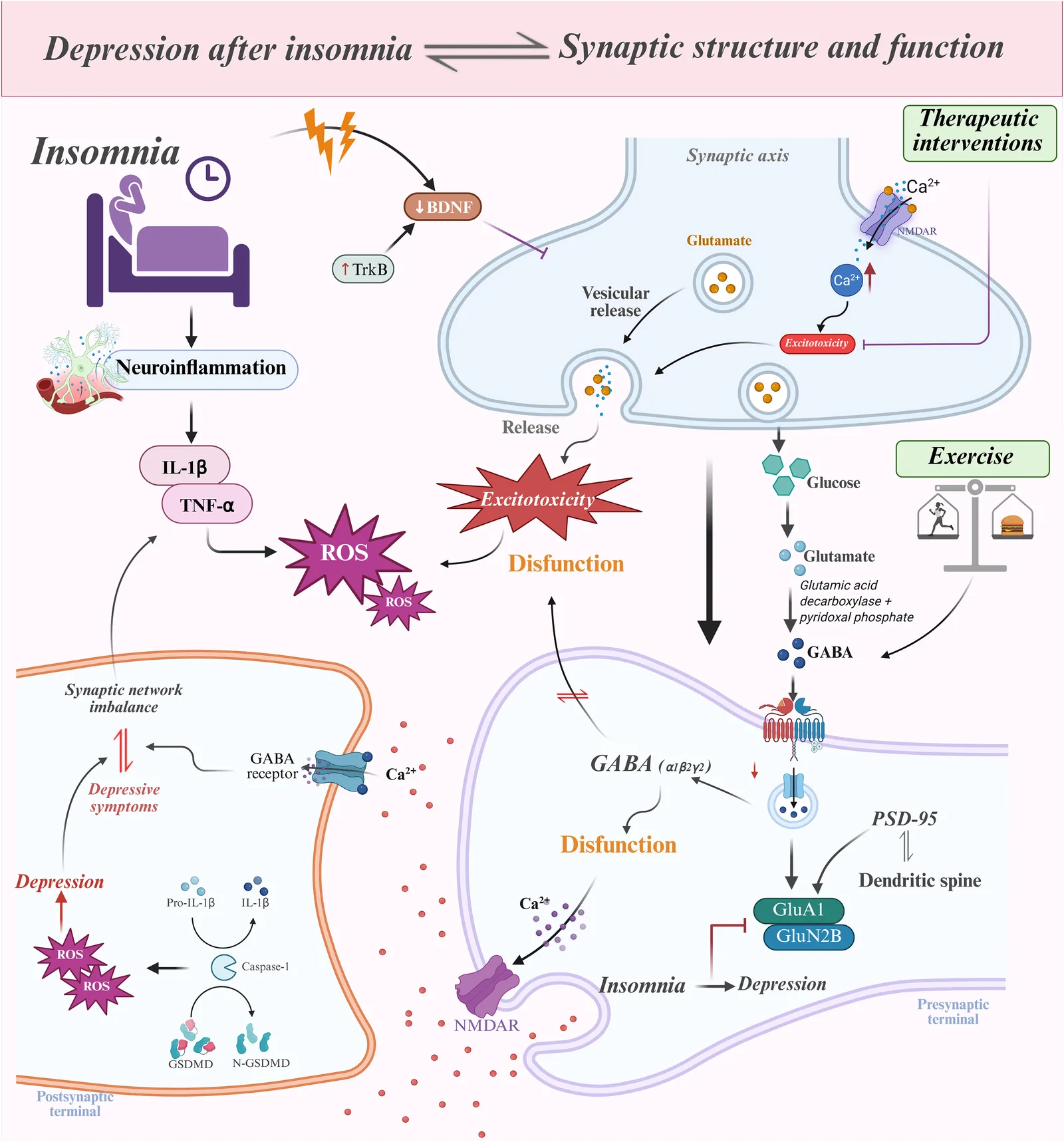
Synaptic axis in insomnia-related depression. Insomnia-induced neuroinflammation and ROS accumulation lead to glutamate excitotoxicity and Ca^2+^ overload, causing dysregulation of NMDAR and AMPAR subunits including NR2B and GluA1, together with impaired GABA_A receptor signaling. These disturbances reduce PSD-95 expression and dendritic spine plasticity. NLRP3/Caspase-1/GSDMD signaling drives synaptic pore formation, while disruption of BDNF/TrkB pathways further destabilizes synaptic networks, collectively resulting in depressive phenotypes. Exercise, calcium channel modulation and enhancement of GABA synthesis show potential to reverse these changes.

### 4.2 The potential application of synaptic plasticity in the treatment of insomnia and depression

Synaptic plasticity restoration has emerged as a central therapeutic strategy and has been extensively investigate. Traditional antidepressants, such as selective 5-HT reuptake inhibitors (SSRIs) and 5-HT-norepinephrine reuptake inhibitors (SNRIs) (94), exert their effects by inhibiting the reuptake of 5-HT and norepinephrine, thereby increasing neurotransmitter concentrations within the synaptic cleft and enhancing neuronal communication (95).

Reports indicate that SSRIs can activate the cAMP/PKA/CREB signaling pathway to upregulate BDNF expression, thereby promoting synaptogenesis and stabilization, and restoring synaptic structure and functional plasticity (96). Targeted drugs aimed at repairing synaptic plasticity show promising therapeutic potential. For instance, the natural compound kaempferol (KMP) has been found to inhibit microglial activation and inflammatory cytokine release, improving hippocampal synaptic architecture and behavioral phenotypes, suggesting an antidepressant effect via an “anti-inflammatory and pro-plasticity” mechanism (97). Traditional Chinese medicine formulas like Tianwang Buxin Pill exert antidepressant effects through sleep promotion, calming the mind, and liver soothing (98).

Non-pharmacological interventions, such as aerobic exercise, also demonstrate clear benefits in enhancing sleep quality and alleviating depressive symptoms. Exercise upregulates hippocampal BDNF protein expression, fostering synaptogenesis and enhancing central signal processing efficiency (99). Moreover, electroacupuncture therapy (4), validated in both clinical and animal studies (100), improves postsynaptic signal transmission and promotes synaptic structural remodeling by upregulating key proteins including NMDAR and PSD-95, thereby improving sleep quality and emotional disturbances (101). Future research integrating neuroimaging, multi-omics, and behavioral assessments aims to elucidate the precise regulatory mechanisms of various interventions on synaptic plasticity, facilitating their translational application in the prevention and treatment of insomnia-associated depression.

## 5 Current status of antidepressant uses and treatment differences for comorbid insomnia

Pharmacological interventions remain the first-line treatment for insomnia-induced depression. However, their long-term efficacy and safety are hindered by several challenges, including frequent adverse effects, development of tolerance, poor patient adherence, and substantial interindividual variability in therapeutic response (102). Currently, antidepressants remain the most commonly prescribed pharmacological agents for treating insomnia-associated depression, primarily including selective SSRIs, SNRIs, mirtazapine, and bupropion (103). Although these agents demonstrate clear efficacy in alleviating depressive symptoms, their effects on comorbid insomnia vary considerably.

Studies indicate that SSRIs may prolong sleep onset latency, reduce total sleep time, and even exacerbate insomnia symptoms. In contrast, mirtazapine and bupropion, due to their sedative properties (104), demonstrate certain advantages in improving sleep quality. However, their use warrants caution because of potential adverse effects such as weight gain, metabolic disturbances, and sexual dysfunction (105). In clinical practice, antidepressants are not routinely employed as first-line treatment for acute insomnia; they are considered alternative options primarily when cognitive behavioral therapy for insomnia (CBT-I) or conventional sedative-hypnotics prove ineffective (106). Therefore, antidepressants are generally utilized as adjunctive or second-line therapies in insomnia management rather than as primary treatment choices.

## 6 Therapeutic interventions for insomnia-associated depression and translational challenges

Adherence issues represent a common barrier in long-term antidepressant therapy, with patients frequently discontinuing medication prematurely due to delayed onset of therapeutic effects (107). Concurrently, adverse effects such as nausea, dry mouth, constipation, and weight gain can further diminish patient satisfaction and adherence (108). Significant interindividual variability in genetic background, drug metabolism capacity, and clinical phenotypes contributes to pronounced heterogeneity in treatment response to the same medication among different patients.

A subset of patients experiences significant therapeutic benefits, whereas others may exhibit suboptimal efficacy or pronounced adverse effects (109). This individualized variability markedly increases the complexity and resource demands of formulating precise clinical treatment strategies. Furthermore, depression patients often present with multiple comorbid somatic conditions requiring polypharmacy, which heightens the risk of drug-drug interactions that can both attenuate therapeutic efficacy and exacerbate adverse reactions (110). Pharmacological treatment planning requires comprehensive evaluation of medication history and interaction risks. While pharmacotherapy is crucial for insomnia-associated depression and may achieve notable short-term benefits, key challenges persist regarding long-term efficacy, safety, adherence, and individual variability (111). Addressing these limitations can optimize treatment strategies and guide personalized interventions and future research.

### 6.1 Pharmacological interventions

Cognitive Behavioral Therapy (CBT) is widely recommended by multiple guidelines as a first-line psychological intervention. However, significant challenges remain in its clinical implementation and dissemination (112). Primarily, limited healthcare resources and a shortage of trained psychotherapists restrict the number of patients who can receive comprehensive CBT (113). Additionally, the time commitment and financial costs associated with multiple therapy sessions further hinder widespread adoption. In current clinical practice, CBT protocols largely rely on standardized templates, which inadequately accommodate the individual characteristics and dynamic psychological states of patients with comorbid depression (114). Moreover, there is a lack of clear consensus and evidence-based guidance tailored to this population.

Moreover, the limited number of therapists with specialized CBT training, coupled with their uneven geographic distribution, further exacerbates disparities in service accessibility. The effectiveness of psychotherapy heavily depends on patients’ subjective initiative and sustained engagement, which are commonly impaired in individuals with depression (115). Symptoms such as diminished motivation, attentional deficits, avoidance behaviors, and negatively biased cognition can all undermine treatment adherence and participation, ultimately affecting therapeutic outcomes. Therefore, despite CBT’s promising theoretical and empirical foundation, its high resource demands, technical complexity, and challenges related to individual compliance hinder standardized and large-scale implementation (116). Further optimization of intervention protocols and dissemination strategies is essential to enhance its practical feasibility and equitable clinical accessibility.

### 6.2 Gut microbiota-targeted interventions: therapeutic potential and clinical considerations

In recent years, the application of gut microbiota modulators such as probiotics, prebiotics, and synbiotics in comorbid conditions has garnered significant attention. Probiotics, as live microorganisms, have certain strains demonstrated to possess antidepressant and sleep-promoting properties. *Bifidobacterium* breve CCFM1025 significantly alleviates depressive behaviors by upregulating BDNF and suppressing pro-inflammatory cytokines IL-6 and TNF-α (117). *Lactobacillus* plantarum JYLP-326 regulates neurotransmitter balance by activating tryptophan hydroxylase 2 (TPH2) (10), thereby enhancing hippocampal 5-HT synthesis. *Lactobacillus brevis* M2, used in insomnia interventions (118), produces fermentation metabolites that convert into gamma-aminobutyric acid (GABA); dose-dependent activation of GABA receptors subsequently prolongs slow-wave sleep and reduces sleep latency. *Lactobacillus fermentum* PS150 improves sleep architecture by inhibiting hyperactivation of the HPA axis (119), thereby lowering corticosterone levels. Clinical evidence indicates that multi-strain formulations containing both *Bifidobacterium* and *Lactobacillus* strains reduce Hamilton Depression Rating Scale (HAM-D) scores, with more stable effects compared to single-strain interventions (120).

Mechanistic studies have revealed that *Bifidobacterium breve* CCFM1025 can enhance the production of SCFAs, increase levels of the 5-HT precursor 5-HTP, and elevate hippocampal synaptic 5-HT concentration (121). Probiotics are generally regarded as safe; however, excessive intake or improper use may cause side effects such as bloating, diarrhea, or abdominal discomfort (122). These adverse effects are typically transient and subside as the body adapts to probiotic supplementation. Nonetheless, in certain high-risk populations-such as immunocompromised individuals, patients with severe illnesses, or recent organ transplant recipients-the use of probiotics may increase the risk of infections (123). Despite promising mechanistic and preclinical findings, the clinical translation of microbiota-targeted interventions remains challenging due to strain-specific effects, heterogeneity in microbial signatures, limited clinical validation, and complex host- microbiota interactions. Recent reviews have highlighted the current evidence status of microbiota-based strategies for psychiatric disorders and emphasized the need for rigorous clinical studies and personalized intervention approaches to improve their therapeutic applicability (124). Future studies should optimize individualized microbial and nutritional interventions to improve therapeutic outcomes in insomnia-associated depression.

### 6.3 Prebiotics and postbiotics: emerging microbiota-based strategies for insomnia-associated depression

Prebiotics, as selective substrates for gut microbiota, include representative compounds such as galacto-oligosaccharides (GOS) and fructo-oligosaccharides (FOS) (125). Studies have shown that prebiotic supplementation containing GOS can modulate gut microbiota composition. However, current clinical evidence remains insufficient to demonstrate consistent improvements in stress-related biomarkers, cortisol awakening response (CAR), or mental health outcomes (126). Therefore, the potential effects of GOS on emotional regulation and stress responses require further validation in well-designed clinical studies.

*Lactobacillus rhamnosus* plus FOS alleviates coronary artery disease with comorbid depression, significantly lowering BDI scores, LPS, and hs-CRP levels, outperforming monotherapy (127). Postbiotics, defined as non-viable microbial products, also exhibit significant bioactivity. For example, cell lysates of *Lactobacillus paracasei* have been confirmed to cross the intestinal barrier and, via the vagus nerve, upregulate *N-acetylserotonin*, thereby shortening sleep latency and enhancing sleep efficiency (128). Their heat stability supports functional food potential. Further details on gut modulators are summarized in **Table 3**. Gut microbiota modulators act by reshaping microbial diversity, enhancing gut-brain barrier integrity, balancing neurotransmitters, and reducing inflammation. Future studies should optimize personalized strain combinations to improve clinical outcomes in comorbid insomnia and depression.

**Table 3:** Microbiota that regulates sleep and improves depression.

Category	Representative bacteria	Function	Pathways	Key targets	Model	Evidence	References
Probiotic	*Lactobacillus rhamnosus*	↑ GABA expression	GABA	GABA receptor	Mouse model	PE	(129)
Probiotic	*Bifidobacterium longum*	↑ 5-HT synthesis, ↓ depressant	Tryptophan, 5-HT	TPH/serotonergic pathways	Adults with IBS	RCE	(130)
Probiotic	*Lactobacillus plantarum*	↑ Sleep, ↓ inflammatory	HPA axis	CRH, ACTH, GR	CUMS rats	PE	(131)
Opportunistic pathogen	*Desulfovibrio spp*	Damages intestinal barrier, ↑ inflammatory	Gut barrier	ZO-1, Occludin	Patients with postpartum depression	OHE	(132)
Opportunistic pathogen	*Prevotella spp*	↑ indole metabolites, ↑ AhR	AhR	AhR	Mechanistic review	PM	(133)
Probiotic	*Faecalibacterium prausnitzii*	↓ inflammatory, ↑ SCFA	SCFA	GPR43	Patients with ulcerative colitis	OHE	(134)
Opportunistic pathogen	*Clostridium spp*	↓ 5-HT, ↑ mood regulation	Tryptophan metabolism	TPH, SERT	Antibiotic induced dysbiosis in mice	PE	(125)
Probiotic	*Akkermansia muciniphila*	↑ intestinal health, ↓ inflammation	Mucosal barrier regulation	MUC2, TFF3	Colitis mice	PE	(135)
Opportunistic pathogen	*Escherichia coli*	↑ Triggers immune, ↑ LPS	TLR4/NF-κB	TLR4, NF-κB	*E. coli* infection in mice	PE	(136)
Probiotic	*Roseburia spp*	↑ butyrate, ↓ inflammation	SCFA-GPR41/43	GPR41, GPR43	Mechanistic review	PM	(137)
Probiotic	*Parabacteroides distasonis*	↓ bile acids, ↓ inflammation	HCA17/ketoDCA	TrkB/BDNF	CUS mice	PE	(138)
Probiotic	*Streptococcus thermophilus*	↑Neurotransmitter metabolism	Dopamine, 5-HT modulation	TPH, SERT, DAT	PCPA induced insomnia mice	PE	(128)
Probiotic	*Eubacterium rectale*	↑ Butyrate, ↓ inflammation	NF-κB	MyD88, TLR4	Human gastrointestinal lymphoma samples and mouse model	PE/OHE	(139)

PE, preclinical evidence; OHE, observational human evidence; RCE, randomized clinical evidence; H/PM, hypothesis or proposed mechanism. ↑, indicates promote/increase; ↓, indicates inhibition/decrease.

### 6.4 Immune-targeted therapies: modulating neuroinflammation and immune dysregulation in insomnia-associated depression

Immune dysregulation drives insomnia-associated depression, marked by elevated pro-inflammatory cytokines and reduced anti-inflammatory (140). This disrupts tryptophan metabolism, impairing 5-HT synthesis and dysregulating NE/DA pathways, which reduces neuroplasticity, dysregulates the HPA axis, and disturbs sleep. These effects create a vicious cycle linking poor sleep and depression (141). IL-2 and IL-15 improve IAD by activating T cells, enhancing anti-inflammatory responses, and restoring neurotransmitter balance. Aspirin and ibuprofen inhibit COX, decreasing IL-1β, IL-6 and TNF-α, alleviating neuroinflammation, and boosting 5-HT and SSRI efficacy (142). Glucocorticoids stabilize the HPA axis, lowering cortisol, reducing inflammation, protecting neurons, and improving mood and sleep (69). Clinical trials demonstrate that anti-inflammatory biologics (tocilizumab, etanercept, infliximab) lower depression scores by blocking inflammatory signaling and restoring neurotransmitter balance (143, 144). Immune pathways thus represent crucial IAD treatment targets. Combining precise anti-inflammatory modulation with personalized non-drug therapies may optimize IAD management. Detailed information on drugs that improve depression by acting on the immune axis is shown in **Table 4**.

**Table 4:** Drugs and compounds targeting the immune axis in depression.

Intervention	Therapeutic effects	Major pathway/targets	Model and regime	Ref.
Fluoxetine	Reduced depressive-like behavior and A1 astrocyte activation	5-HT2BR/β-arrestin2	CMS mice; 10 mg/kg/day, i.p., 4 weeks	(145)
Minocycline	Reduced depressive-like behavior and neuroinflammation	Microglial polarization; BDNF/TrkB	CUMS rats; 40 mg/kg/day, 45 days	(146)
Astragaloside IV	Reduced depressive-like behavior and hippocampal inflammation	PPARγ/NF-κB/NLRP3	Restraint-stress mice; 16–64 mg/kg/day, i.g	(147)
Baicalin	Reduced depressive-like behavior and neuroinflammation	PI3K/AKT/FoxO1; TLR4	CUMS mice; 30–60 mg/kg/day, oral, 5 weeks	(148)
Gypenosides	Reduced depressive-like behavior and microglial activation	IKKα/IKKβ/NF-κB	CUMS mice; 100 mg/kg/day, oral, 4 weeks	(149)
Safflower extract	Reduced depressive-like behavior and inflammatory cytokines	TLR4/p38/NF-κB/NLRP3	CUMS mice; 10–30 mg/kg/day, oral, 4 weeks	(150)
Brilliant Blue G	Reduced LPS-induced depressive-like behavior and TNF-α	P2X7R	LPS mice; 50 mg/kg, i.p., before LPS	(151)
Melatonin	Reduced LPS-induced depressive-like behavior and neuroinflammation	TNF-α; BDNF; oxidative stress	LPS mice; 10 mg/kg, i.p., before LPS	(152)

CMS, chronic mild stress; MDD, major depressive disorder; i.p., intraperitoneal; i.g., intragastric; 5-HT2BR, serotonin 2B receptor; P2X7R, P2X7 receptor; PPARγ, peroxisome proliferator-activated receptor γ. Brilliant Blue G (BBG) is included as an experimental P2X7R antagonist and mechanistic tool compound rather than an established clinical therapeutic.

## 7 Traditional Chinese medicine formulations for treating insomnia-associated depression

Traditional Chinese Medicine (TCM) employs multi-herb formulas based on the “Jun-Chen-Zuo-Shi” principle to maximize efficacy and minimize toxicity (153). These formulas act through multiple components and pathways, matching disease complexity. For instance, Xiaoyao San (containing Chaihu, Baishao, Danggui, etc.) has shown potential benefits for insomnia-associated depression in the context of liver qi stagnation (154). Pharmacological studies suggest that Xiaoyao San may alleviate depression through modulation of the HPA axis and microbiota-gut-brain axis, potentially involving neuroendocrine and neuroimmune pathways. Enhanced formulations like Jieyu Pill (with Curcuma, Albizia bark, and Gancao-Maidazao) may further regulate liver qi and heart-mind harmony, with reported benefits for sleep disturbances, anxiety, palpitations, and autonomic dysfunction (155). For heart-spleen deficiency, Guipi Decoction is traditionally used to replenish Qi, fortify the spleen, nourish blood, and calm the mind, with reported effects on mood, fatigue, insomnia, and memory (156). The reported effects and proposed mechanisms of Chinese herbal formulas for insomnia-associated depression are summarized in **Table 5**.

**Table 5:** Improving insomnia-related depression with Chinese herbal medicine compounds.

Name	Effects	Nature/flavor	Meridians	Pathways	Targets	References
*Xiaoyao San*	Soothe liver, regulate Qi, relieve depressive insomnia	Sweet, slightly bitter	Liver, Spleen	PI3K-Akt, HPA axis, microbiota-gut-brain axis	AKT1, TP53, VEGFA, 5‑HT	(157)
*Jiawei Xiaoyao San*	Enhanced antidepressant effect, improve sleep	Slightly sweet, bitter	Liver, Spleen	HPA axis, neurotrophic	BDNF, CORT, 5‑HT	(158)
*Jieyu Wan*	Soothe liver and heart, relieve anxiety and palpitations	Pungent, sweet	Liver, Heart	Monoamine neurotransmitter	5‑HT, DA	(159)
*Guipi Tang & Guipi Wan*	Tonify Qi and blood, calm mind, treat depressive insomnia	Sweet, warm	Heart, Spleen	Immunomodulation, neurotransmitter balance	BDNF, neurotrophic factors	(160)
*Suanzaoren Tang*	Tranquilize mind, improve sleep with irritability	Sweet, sour, slightly warm	Heart, Liver	GABAergic, serotonergic	GABA, 5‑HT	(161)
*Kaixin San*	Nourish heart and spleen, relieve depressive insomnia	Sweet, mild	Heart, Spleen	Synaptic, monoamine	GABA, monoamines, neurotrophic proteins	(162)
*Chaihu Longgu Muli Tang*	Regulate liver Qi, sedate heart and mind	Bitter, sweet, neutral	Liver, Heart	HPA axis	CORT, HPA axis proteins	(163)
*Guizhi Gancao Longgu Muli Tang*	Calm spirit, relieve palpitations and insomnia	Sweet, pungent, neutral	Heart, Lung, Heart	Central nervous system	Autonomic regulators	(164)
*Xiaochaihu Tang*	Harmonize Shaoyang, relieve emotional imbalance	Bitter, slightly cold	Liver, Gallbladder	Monoaminergic neurotransmission	NE, 5‑HT	(165)
*Baihe Dihuang Tang*	Anti-depressive, anxiolytic, neuroprotective	Sweet, cool	Heart, Lung, Kidney	Inflammatory regulation, epigenetic neuroprotection	CORT, ILs, HPA factors, miRNA-mRNA networks	(166)
*Sinisan*	Soothe liver, harmonize spleen, improve sleep	Bitter, sweet, neutral	Liver, Spleen	Apoptosis, neurotransmitter modulation	CREB1, NOS3, CASP3, SLC6A4	(94)

Analysis of China’s patent data reveals the top 10 anti-depression herbs: turmeric, bupleurum, white peony, sour jujube seed, poria, angelica, polygala, atractylodes, acorus, and salvia. These herbs are traditionally used for patterns involving qi stagnation, blood stasis, and deficiency, primarily affecting the liver, heart, spleen, and lungs (157). Their traditional functions include liver-soothing, spleen-heart fortification, and qi and blood regulation, offering insights for further research on depression treatment and drug development.

## 8 Discussion

Insomnia is closely associated with the onset, persistence, and recurrence of depression, yet the biological basis of this comorbidity cannot be fully explained by circadian disruption alone. The evidence reviewed here supports a broader model in which sleep loss alters microbial composition and function, weakens intestinal barrier integrity, and changes the production of immunoregulatory metabolites. Current evidence suggests that sleep disruption is associated with alterations in microbial composition and function, although a consistent microbial signature of insomnia associated depression has not yet been established. Altered microbial metabolism may affect tryptophan availability, kynurenine pathway activity, serotonin related signaling, and the production of immunoregulatory metabolites, thereby influencing neuroimmune and synaptic processes relevant to mood regulation. The main value of the microbiota immune synapse framework lies in connecting peripheral disturbances induced by insomnia with central changes relevant to depression, rather than treating microbial dysbiosis, inflammation, and synaptic dysfunction as independent abnormalities.

Repeated sleep disruption may produce sustained or dysregulated HPA axis activity, although cortisol responses vary according to insomnia phenotype, sampling time, disease duration, and comorbid conditions. HPA axis dysregulation, microbial metabolite depletion, and epithelial stress may jointly weaken tight junction integrity and increase exposure to microbial products such as lipopolysaccharide. These signals can activate innate immune pathways, including TLR4 and NF κB signaling, and promote the release of IL 1β, IL 6, and TNF α. These mediators may affect the brain through humoral, neural, and cellular routes, alter blood brain barrier function, activate microglia and astrocytes, and interfere with neurotrophic signaling and synaptic remodeling. This interpretation places immune dysregulation at the interface between microbial disturbance and synaptic injury and provides a mechanistic basis for evaluating nutritional interventions.

The HPA axis and vagal pathways may reinforce this network by coupling sleep and stress signals with intestinal, microbial, and immune responses. This network is likely to operate bidirectionally, because inflammation and altered microbial metabolism may also disturb sleep architecture and stress regulation, thereby maintaining depressive symptoms. Microbiota transfer experiments from sleep deprived donors provide evidence that microbial alterations can contribute to behavioral and neuroimmune abnormalities in recipient animals. Accordingly, the microbiota immune synapse model proposed in this review should be interpreted as an integrative framework rather than a fully validated causal pathway in humans. These findings support causality at the preclinical level but do not establish that gut dysbiosis alone causes clinically diagnosed insomnia associated depression in humans. Current treatments, including cognitive behavioral therapy for insomnia, pharmacological agents, light-based interventions, and selected nutritional approaches, can improve sleep or mood outcomes. Evidence that these treatments fail because they target isolated pathways is limited, and several may already influence immune or microbial processes indirectly. The more relevant limitation is that few clinical trials have simultaneously assessed microbial function, immune activity, synaptic indicators, sleep outcomes, and depressive symptoms. Mechanistic validation is particularly limited in human comorbidity cohorts, and reliable biomarkers for selecting patients or predicting treatment response have not been established.

The principal contribution of this review is the integration of three levels of pathology that are often examined separately: insomnia associated microbial dysfunction, peripheral and central immune dysregulation, and synaptic impairment. Within this framework, immune signaling is not treated as one pathway among many but as the mechanistic interface that translates intestinal disturbance into altered neural plasticity. This framework supports the evaluation of microbiota targeted nutritional interventions as adjunctive, mechanism informed strategies. Their potential advantage lies in acting on several modifiable components of the proposed loop, including microbial metabolism, epithelial barrier integrity, inflammatory signaling, and synaptic support. Their clinical value will depend on identifying responsive patient subgroups and demonstrating that changes in these mechanisms mediate symptom improvement.

Compared with existing literature, this review brings molecular signaling, immune inflammation, and synaptic dysfunction into a microbiota immune synapse framework. Within this framework, immune dysregulation may serve as an important link between insomnia related gut microbial changes, impaired synaptic plasticity, and depressive symptoms. These processes may further interact to form a pathological cycle involving sleep disturbance, microbial imbalance, inflammation, and synaptic injury. This integrated view may provide a clearer basis for developing nutritional strategies that act on several related pathways rather than a single target.

Future studies may benefit from combining metagenomics, metabolomics, immune profiling, and sleep assessment to clarify the temporal sequence of microbiota immune neural changes. Longitudinal designs could help distinguish early contributors from secondary alterations. Repeated measurements of microbial metabolites, intestinal barrier markers, inflammatory mediators, and sleep parameters may also improve the evaluation of nutritional interventions. Attention to sampling time is important because microbial metabolism, cortisol secretion, and inflammatory responses can vary across circadian cycles. Clinical translation will likely depend on standardized protocols and well-designed trials that assess sleep, depressive symptoms, microbial function, and immune outcomes in an integrated manner.

## 9 Conclusion

The microbiota immune synapse axis provides an integrative framework for understanding insomnia related depression and may guide the development of food derived bioactive compounds and precision nutritional strategies targeting microbial dysbiosis, immune imbalance, and synaptic dysfunction.
